# Enhanced Osteogenic Differentiation in Zoledronate-Treated Osteoporotic Patients

**DOI:** 10.3390/ijms18061261

**Published:** 2017-06-13

**Authors:** Luca Dalle Carbonare, Monica Mottes, Giovanni Malerba, Antonio Mori, Martina Zaninotto, Mario Plebani, Alessandra Dellantonio, Maria Teresa Valenti

**Affiliations:** 1 Department of Medicine, Internal Medicine, Section D, University of Verona, Piazzale Scuro, 10, 37134 Verona, Italy; luca.dallecarbonare@univr.it (L.D.C.); antonio.mori@univr.it (A.M.); alessandra.dellantonio@univr.it (A.D.); 2 Department of Neurosciences, Biomedicine and Movement Sciences, University of Verona, Piazzale Scuro, 10, 37134 Verona, Italy; monica.mottes@univr.it (M.M.); giovanni.malerba@univr.it (G.M.); 3 Department of Laboratory Medicine Azienda Ospedaliera, University of Padova Via Giustiniani, 2, 35128 Padova, Italy; martina.zaninotto@unipd.it (M.Z.); mario.plebani@unipd.it (M.P.)

**Keywords:** mesenchymal stem cells, Runt-related transcription factor 2 (*RUNX2*), zoledronic acid, gene expression, bone turnover, differentiation

## Abstract

Bisphosphonates are well known inhibitors of osteoclast activity and thus may be employed to influence osteoblast activity. The present study was designed to evaluate the in vivo effects of zoledronic acid (ZA) on the proliferation and osteoblastic commitment of mesenchymal stem cells (MSC) in osteoporotic patients. We studied 22 postmenopausal osteoporotic patients. Densitometric, biochemical, cellular and molecular data were collected before as well as after 6 and 12 months of ZA treatment. Peripheral blood MSC-like cells were quantified by colony-forming unit fibroblastic assay; their osteogenic differentiation potential was evaluated after 3 and 7 days of induction, respectively. Circulating MSCs showed significantly increased expression levels of osteoblastic marker genes such as Runt-related transcription factor 2 (*RUNX2*), and Osteonectin (*SPARC*) during the 12 months of monitoring time. Lumbar bone mineral density (BMD) variation and SPARC gene expression correlated positively. Bone turnover marker levels were significantly lowered after ZA treatment; the effect was more pronounced for C terminal telopeptide (CTX) than for Procollagen Type 1 N-Terminal Propeptide (P1NP) and bone alkaline phosphatase (bALP). Our findings suggest a discrete anabolic activity supported by osteogenic commitment of MSCs, consequent to ZA treatment. We confirm its anabolic effects in vivo on osteogenic precursors.

## 1. Introduction

Osteoporosis is a degenerative disease associated with bone fractures affecting mobility and increasing mortality and morbidity in older populations [1]. Bone resorption is associated with osteoclastic activity, and increases in postmenopausal osteoporosis leading to an imbalance in bone homeostasis. However, the bone loss observed with aging is not only associated with the increased activity of osteoclasts, but also with a progressive decline in osteoblast number, function, and survival that is concurrent to the generation of bone formation impairment [2]. The ability to produce an adequate number of functional osteoblasts is pivotal for bone mass preservation. With regard to this aspect, it has been demonstrated that bone remodeling involves the proliferation and osteogenic differentiation of mesenchymal stem cells [3]. Mesenchymal stem cells (MSC) can also differentiate into adipocytes; a mutually inhibitory relationship exists between osteogenic and adipogenic lineage commitment [4].

According to this observation, histomorphometric analyses revealed adipocytic replacement in the bone marrow of postmenopausal osteoporotic patients [5]; furthermore, we have demonstrated in a previous study that mesenchymal stem cells of osteoporotic patients have an abnormal osteogenic differentiation [6]. Recently, we also demonstrated that the osteogenic master factor Runt-related transcription factor 2 (*RUNX2*) downregulation and the adipogenic master gene Peroxisome Proliferator Activated Receptor Gamma *PPARG2* upregulation in osteoporotic patients may be enhanced by modified lipoproteins derived from the oxidation of arachidonate-containing phospholipids (ox-PAPCs) [7]. This imbalance between osteoblastogenesis and adipogenesis has been considered in order to develop therapies capable of promoting osteoblastogenesis or inhibiting adipogenesis [8], although current treatments for osteoporosis are still mainly represented by drugs with antiresorptive effects such as bisphosphonates (BPs). Bisphosphonates are analogues of pyrophosphate with a high affinity to hydroxyapatite crystals that increases their efficacy in metabolic bone diseases. They maintain bone strength further than what is expected on the basis of the moderate bone mineral density increase that they induce.

Even if BPs are considered antiresorptive drugs, this mode of action cannot fully account for their efficacy on fracture reduction. Many studies suggest that they can also promote osteogenesis through different mechanisms involving mesenchymal stromal cell differentiation [9,10,11] and/or reduction of bone marrow fat [12]. In this regard, it has been observed that in vitro short-term exposure to zoledronic acid (ZA) enhanced osteogenic differentiation and mineralization of mesenchymal stem cells, suggesting a direct positive effect of BPs on osteogenic precursors [13]. There is now a growing body of evidence suggesting that the preventive effects of BPs on bone loss may also be ascribed to their anabolic activity on cells of the osteogenic lineage. BPs control osteoblastic functions such as proliferation and differentiation [14], prevent osteoblast apoptosis [15], modulate osteoblastic production of extracellular matrix proteins [16,17], and also regulate osteoblastic expression and secretion of various growth factors and cytokines [18,19]. Thus, it has been hypothesized that osteoblasts are the complementary elements required for the complete antiresorptive effects of these drugs [20].

Since the dynamic equilibrium of bone turnover is maintained by the commitment of MSCs as well as by osteoclastic activity, it is interesting to analyze the modulation of both cell types after exposure to BP treatment. Many studies on the effects of BPs on osteoclasts have been reported [21]. Despite the combination of bone resorption and bone formation, the effects of BPs on MSCs have not been investigated in treated osteoporotic patients so far. Effectively, while in vitro studies have suggested a direct effect of BPs on osteogenesis, few studies have been performed by stimulating in vitro MSCs harvested from patients [22]. Thus, the in vivo modulation of MSCs in BP-treated patients has not yet been explained. Data obtained from weekly ZA-treated rats demonstrated that treatment stimulates the ex vivo proliferation of their MSCs, but has no effect on osteoblastic differentiation [23].

In this study, we describe for the first time the ex vivo effects on MSCs isolated from osteoporotic patients after a single infusion of ZA in terms of proliferation and osteogenic gene expression. We also provide data showing the relationship between molecular osteogenic levels, bone mineral density, and bone turnover markers.

## 2. Results

**Figure 1:**
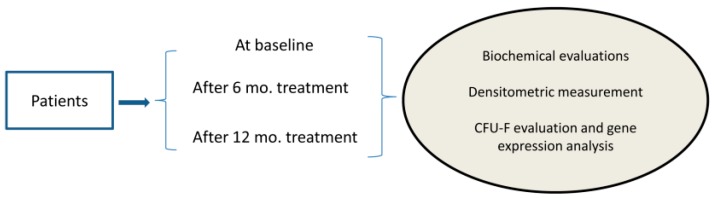
Schematic representation of the experimental design.

The experimental design is represented in Figure 1. The main characteristics of the study population at the baseline and 12 months after treatment are reported in Table 1.

### 2.1. Densitometric and Biochemical Evaluation

All patients showed bone densitometric values lower than −2.5 SD at the lumbar spine or other skeletal sites, according to the inclusion criteria.

At the baseline, five patients out of 22 showed 25-hydroxy (25-OH)–vitamin D levels below the normal values, while all patients presented normal values of parathormon (PTH), C terminal telopeptide (CTX), and other metabolic parameters. After cholecalciferol supplementation, all patients were in the normal vitamin D range; vitamin D levels did not change during the study. In the study group, after ZA treatment, we observed a significant reduction of serum calcium and calcium excretion levels with a secondary significant increase of PTH.

### 2.2. Bone Turnover Markers

**Figure 2:**
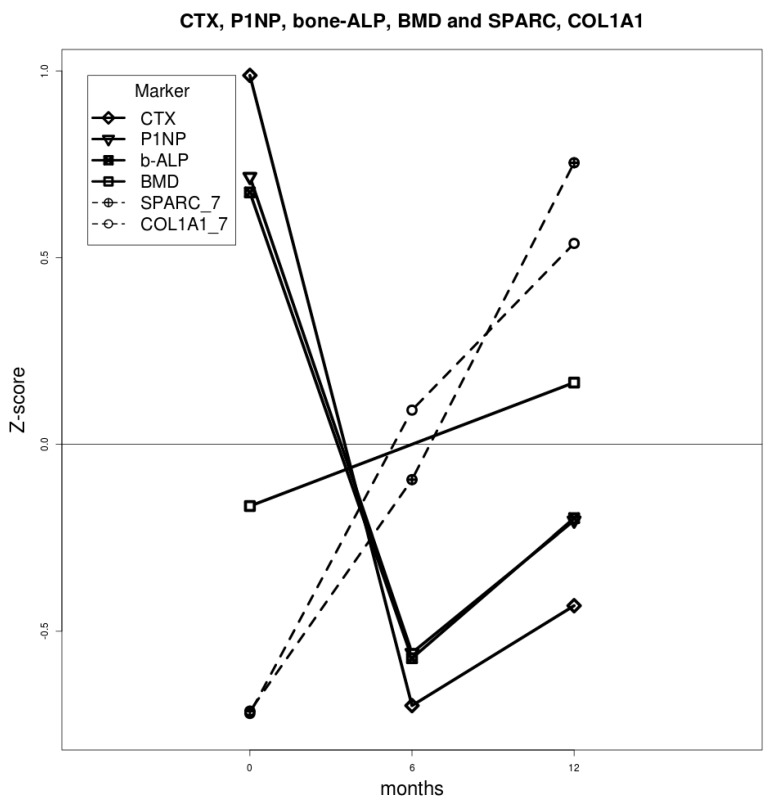
Mean values of bone metabolism/osteogenic differentiation markers across the time points. Biochemical values were obtained from blood samples; qRT-PCR values obtained from differentiating cells were utilized as gene expression markers. For each marker, mean values were calculated from the standardized z-score. The lumbar BMD increase during treatment correlated significantly with the expression of osteogenic marker *SPARC* (*p* < 0.05); *COL1A1* gene expression showed an analogous trend without reaching any statistical significance (*p* > 0.05).

We observed a significant increase of bone mineral density (BMD) 12 months after ZA infusion. Consistently, we observed a decrease in bone turnover markers (CTX, P1NP and ALP) evaluated after 6 months of treatment; the decrease was more pronounced for CTX than for P1NP and bALP. Both turnover marker values rose between 6 and 12 months, although they remained lower than those at baseline. Interestingly, *SPARC* and *COL1A1* gene expression showed the same trend of bone formation markers (P1NP and ALP) between 6 and 12 months (Figure 2).

### 2.3. Colony Forming Unit-Fibroblastic (CFU-F) Evaluation

**Figure 3:**
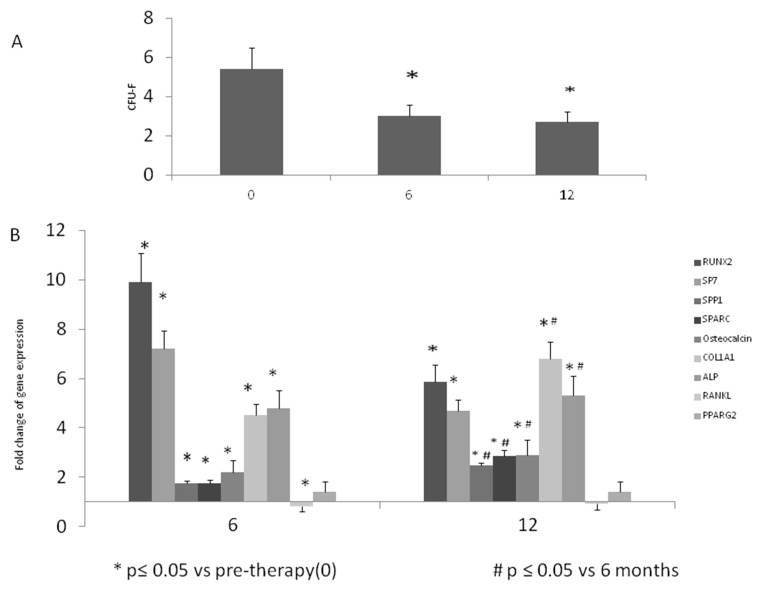
Peripheral blood derived-MSCs and osteogenic differentiation after ZA infusion. Cellular and molecular analyses display the reduction of Colony Forming Unit-Fibroblastic (CFU-F) (**A**) and the increase of osteogenic expression (**B**) during the treatment. The values are representative of 22 patients at each observation time. Three independent qRT-PCRs were performed at the same time for each gene. The overall information suggests an enhanced osteoblastogenesis in ZA-treated patients. (* *p* < 0.05 vs. pre-therapy; ^#^ *p* < 0.05 vs. 6 months).

The MSC-like obtained by the depletion method of mononuclear cells (MNCs) from the controls and patients expressed cluster differentiation markers at similar levels (Table 2). After 14 days of cultivation, we assayed CFU-F and a higher number was observed at baseline of treated patients (5.67 ± 1.7) compared to normal donors (2.52 ± 1.69). Therefore, we observed a decreased frequency of CFU-F obtained after 6 (3 ± 1.6) (*p* < 0.05) and 12 months (2.77 ± 1.7) (*p* < 0.05) of ZA infusion to osteoporotic patients (OP) compared to the pre-therapy condition (5.45 ± 2.8) (Figure 3A).

### 2.4. Gene Expression

In order to track the osteogenic gene expression, we evaluated the levels of *RUNX2* after 3 days of differentiation, as this gene is a marker for early commitment of MSCs [24]. *SP7*, *SPP1*, *SPARC*, *COL1A1*, Osteocalcin, and *ALP* marker levels were instead evaluated after 7 days of culture in osteogenic medium, in order to analyze the progression of the phenotype in committed mesenchymal cells. As reported in Figure 3B, *RUNX2* gene expression showed a nearly 10-fold increase after 6 months of treatment; after 12 months, it settled to levels still significantly higher compared to the pre-treatment levels (*p* < 0.05). Also, the expression levels of *SP7*, *SPP1*, *SPARC*, *COL1A1*, *Osteocalcin*, and *ALP* were higher after 6 and 12 months compared to the pre-treatment levels. In addition, we observed a significantly increased expression of *SPP1*, *SPARC*, *Osteocalcin*, *COL1A1*, and *ALP* after 12 months compared to 6 months of treatment (*p* < 0.05). As for the modulation of *RANKL*, we observed a lower gene expression after 6 and 12 months of treatment. This reduction reached a statistical significance after 6 months (*p* < 0.05).

**Figure 4:**
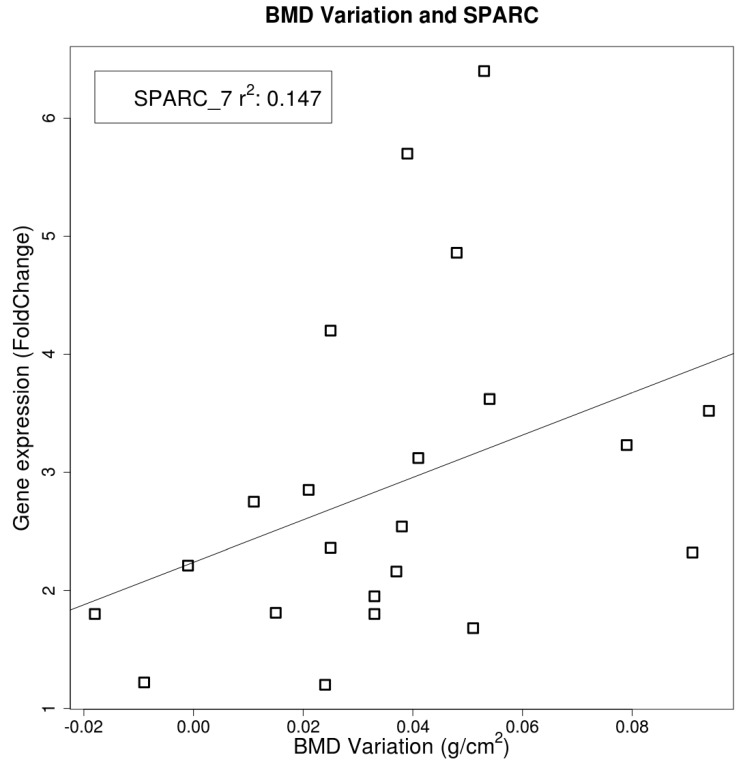
Linear correlation between lumbar spine bone mineral density (BMD) and SPARC gene expression levels (represented as fold change). The correlation was statistically significant (*p* < 0.05).

The increase of BMD at the lumbar spine after ZA infusion correlated positively (*r*^2^ = 0.147) with the expression of *SPARC*, a marker gene of mature osteoblast, evaluated in osteoporotic (OP) patients MSC-like after 7 days of differentiation. (Figure 4). This gene was noted to be a predictor of lumbar bone density by the covariance analysis (*p* < 0.05).

In order to evaluate a possible effect of ZA on the adipogenic lineage, we analyzed *PPARG2* gene expression in MSCs-like after 6 and 12 months of treatment. As reported in Figure 3B, no statistically significant change was observed during treatment, compared to pre-therapy levels.

## 3. Discussion

BPs are considered frontline agents against osteoporosis and are the most prescribed treatments for the prevention of bone loss and related fractures [25,26]. It is known that BPs act mainly by inhibiting osteoclast activity, but growing evidence indicates that they may stimulate osteoblast differentiation as well. We have addressed this issue by monitoring the effects of a single ZA infusion on 22 osteoporotic patients. Biochemical, densitometric, radiologic, and molecular data concerning expression levels of osteogenic marker genes were recorded at baseline as well as 6 and 12 months after treatment. As expected, bone turnover markers levels were significantly lowered after ZA treatment and we also observed a reduction of *RANKL* gene expression in MSCs-like after 6 months of treatment. The effect was more pronounced for CTX than for P1NP and ALP. Likewise, a similar pattern was observed in the second 6-month period following ZA infusion; the difference between levels of bone resorption/bone formation markers was maintained during the study. This last finding suggests a fairly appreciable anabolic activity supported by osteogenic commitment of MSC-like. Despite the inherent limitations of an ex vivo MSC differentiation model, our data suggest that the observed BMD increase in patients after treatment correlates with enhanced osteoblastogenesis, substantiated by the significantly increased expression levels of osteoblastic marker *SPARC*. By comparing the gene expression of peripheral blood derived (PB)-MSCs after 6 and 12 months of ZA infusion, we observed an enhanced osteogenic commitment, and the gene expression levels were higher compared to baseline (before ZA treatment). The most evident increase in expression levels of *RUNX2*, the early osteogenic commitment marker, was monitored in PB-MSCs of patients 6 months after ZA infusion; after 12 months, *RUNX2* gene expression was still significantly higher than at baseline. A similar trend was observed for its downstream transcription factor SP7. The expression of bone extracellular matrix (ECM) markers, such as *COL1A1*, *SPARC*, *Osteocalcin*, and *ALP*, constantly increased during the 12 months of monitoring time, suggesting enhanced bone formation. Lumbar BMD variation and *SPARC* gene expression were positively correlated. In addition, in our study we did not observe any effect of ZA on adipogenic differentiation, evaluated by *PPARG2* gene expression levels in MSCs-like. Levels of PB-MSC of osteoporotic patients, evaluated for their capacity to give rise to fibroblastic colonies (CFU-F), were significantly higher before treatment than after 12 months of treatment. In our experience, levels of circulating MSCs were consistently higher in untreated osteoporotic patients than in normal donors [6]. This may reflect an enhanced mobilization of MSCs to produce osteogenic cells as a consequence of increased bone turnover. Interestingly, values recorded after treatment (mean ± SD, 2.7 ± 1.68) reproduced those observed in PB-MSC of normal donors, suggesting a normalization of bone turnover promoted by ZA.

Our investigation, focusing particularly on the ex vivo effects at the molecular and cellular level of ZA treatment, supports its anabolic effects on osteogenic precursors, previously demonstrated in vitro [27] and in a sickle cell disease mouse model [28].

The relationship between osteogenic gene expression and bone turnover markers supports the hypothesis that the significant increase in bone density after ZA treatment is not only due to the inhibition of bone turnover, but also due to new bone formation as a consequence of the commitment of mesenchymal stem cells through the osteogenic lineage. The stimulation of osteoblasts, following turnover inhibition, shows the potential anabolic activity of ZA, already described to explain the anabolic action of teriparatide [29]. This mode of action might be peculiar to ZA, considering the different bone turnover profiles generated by other BPs, e.g., risedronate, in a specific context [30].

The peculiar activity of ZA demonstrated in this study suggests its employment not only in clinical conditions characterized by increased bone turnover, but also in other disorders, where osteoblastic activity results are affected. This a perspective may explain the powerful effect of ZA in glucocorticoid-induced osteoporosis, a condition characterized by a significant decrease of osteogenic activity, associated with the depletion of osteoblastic precursors and apoptosis of mature cells. The bivalent effect of ZA is of clinical relevance considering a possible modulation in its administration schedule, singly or in association with other drugs, using a sequential approach.

## 4. Materials and Methods

### 4.1. Ethics Statement

Written informed consent was obtained from all participants and the study was approved by the Ethical Committee of Azienda Ospedaliera Universitaria Integrata of Verona, Italy (number 1538; 3th December, 2012; Local Ethical Committee of Azienda Ospedaliera Integrata di Verona).

### 4.2. Patients

We selected 22 postmenopausal OPs who had been consecutively referred to our Metabolic Bone Disease Centre in a 6-month interval (from January to June) of 2012. We compared biochemical and molecular data of these patients before and after 6 and 12 months of ZA treatment. None of the patients or controls were taking any therapy before the enrolment. At baseline, before ZA infusion, patients were supplemented with a bolus of 300.000 UI of cholecalciferol for two days, followed by a monthly dosage of 100.000 UI per os. Exclusion criteria included premature menopause (younger than 45 years old), or presence of a disease or use of a drug that could affect bone or calcium/phosphate metabolism.

At baseline and 12 months after ZA (5 mg, Novartis Basel, Switzerland) infusion, all women included in the study underwent a densitometric (DXA) measurement at the lumbar spine and hip (Hologic QDR Discovery Acclaim, Waltham, MA, USA) and osteoporosis was diagnosed according to World Health Organization (WHO) parameters. A lumbar spine or femoral T score lower than 2.5 SD was considered as the cut-off [31].

Biochemical evaluations were performed on all OPs in order to rule out secondary causes of osteoporosis (*S*-calcium (Ca), *S*-phosphate (P), calcium/creatinine ratio (CaE), Parathyroid Hormone (PTH), carboxy-terminal telopeptide of type I collagen (CTX), alkaline phosphatase (ALP), 25-hydroxyvitamin D (VitD)). In addition, cholesterol levels and CRP (C Reactive Protein) were evaluated. Patients with low levels of vitamin D were supplemented and enrolled when normal levels were reached.

### 4.3. Sample Collection for Serum and Circulant MSCs-Like

The serum was obtained from 10 mL of fresh blood by centrifugation at 400× *g* and frozen at −80 °C until use. MSC-like cells were isolated from 50 mL of heparinised blood using two Ficoll procedures to deplete hematopoietic cells by antibodies cocktail, as previously reported [32]. The enriched cells obtained were washed in phosphate buffered saline (PBS) and analyzed for gene expression.

### 4.4. Analysis of Cell Phenotype

We analyzed gene expression for CD3, CD14, CD19, CD45, and CD34 markers, as previously reported [32]. This method allowed phenotypic analysis in a small number of cells obtained by stringent stem-cell purification strategies, as previously described [33].

### 4.5. Quantification of Human Mesenchymal Progenitors (CFU-F)

To evaluate the number of CFU-F assays, MSC-like were seeded at three different concentrations (4 × 10^5^, 2 × 10^5^, 1 × 10^5^) in 24-well plates and incubated for 14 days in medium containing MesenCult MSC Stimulatory Supplement (#05402, StemCell Technologies Inc., Vancouver, BC, Canada) under a humidified atmosphere of 5% CO_2_ at 37 °C, as previously reported [32]. After 14 days, adherent cells were fixed with methanol and stained for 5 min with Giemsa staining solution. After washing with distilled water, colonies consisting of more than 50 cells were scored on an inverted microscope. The CFU-Fs were expressed as an average of the three different culture concentrations.

### 4.6. Osteogenic Differentiation

The osteogenic differentiation was obtained by using osteogenic medium containing 15% of Osteogenic Stimulatory Supplements (#05404, Stemcell), 10^−8^ M dexamethasone, 3.5 mM β-glycerophosphate, and 50 μg/mL ascorbic acid (StemCell Technologies Inc., Vancouver, BC, Canada), as previously reported [32]. The medium was changed every 3 days after initial plating. After 3, 7, and 14 days of osteogenic induction, cells were harvested and the pellet was processed or stored at −80 °C until use.

### 4.7. Total RNA Extraction

Total RNA was extracted by using the RNAeasy minikit (Quiagen, Hilden, Germany) with DNAse I and analyzed by measuring the absorbance at 260 and 280 nm. RNA integrity was confirmed by RNA electrophoresis on a 1.5% agarose gel containing ethidium bromide.

### 4.8. Reverse Transcription (RT)

First-strand cDNA was obtained by using the First Strand cDNA Synthesis Kit (GE Healthcare, Little Chalfont, UK), with random hexamers (GE Healthcare) according to the manufacturer’s protocol. Reverse Transcription (RT) products were stored at −80 °C.

### 4.9. Real Time PCR

PCRs were performed in a total volume of 25 μL containing Taqman Universal PCRMaster mix, no AmpErase UNG, and 20 ng of cDNA from each sample; pre-designed, gene-specific primers and probe sets for each gene (CD3, Hs00174158_m1; CD14, Hs02621496-s1; CD19, Hs00174333_m1; CD45, Hs00174541_ m1; CD34, HS00156373_m1; RUNX2, Hs00231692_m1; OSTERIX (SP7) Hs00541729_m1; B2M, Hs999999_m1; COLLAGEN, TYPE I, ALPHA 1 (COL1A1) Hs00164004_m1; OSTEONECTIN (SPARC) Hs00234160_m1; OSTEOPONTIN (SPP1) Hs00167093_m1; OSTEOCALCIN (BGLAP) Hs01587813_g1; RANKL (TNFSF11) Hs01092186_m1; *PPARG*2 Hs01115513_m1; GAPDH, 0802021; Applied Biosystems (Thermo Fisher corporation, Waltham, MA, USA); were obtained from Assay-on-Demand Gene Expression Products (Applied Biosystems). Real time RT-PCR reactions were carried out in a two-tube system and in multiplex. The real time amplifications included 10 min at 95 °C (AmpliTaq Gold activation), followed by 40 cycles at 95 °C for 15 s and at 60 °C for 1 min. Thermocycling and signal detection were performed with an ABI Prism 7300 Sequence Detector (Applied Biosystems). Signals were detected according to the manufacturer’s instructions. To evaluate the cell phenotype, mRNA quantification was analyzed by the Relative Standard Curve Method, as previously described [6]. To compare osteogenic mRNA levels during differentiation in MSCs before and after ZA treatment, gene expression was calculated after normalization against the housekeeping genes (B2M, GAPDH) using the relative fold expression differences [34]. *C*_t_ values for each reaction were determined using TaqMan SDS analysis software (7300 System SDS v1.4, Thermo Fisher corporation, Waltham, MA, USA). For each amount of RNA tested triplicate *C*_t_ values were averaged.

### 4.10. Statistical Analysis

Paired *t*-test was used to compare the variation of the same variable at two different time points.

The analysis of covariance (ANCOVA) was used to examine the association of biomarker changes (at different time points) with BMD. The ANCOVA was repeated for different types of biomarkers (e.g., CTX, P1NP and ALP) separately. Association between the variations of two different variables at pairs of time points was estimated through linear.

## 5. Conclusions

In conclusion, we believe that our work confirms the efficacy of ZA in increasing bone mass in osteoporotic patients. This effect is not only associated with a decrease of bone turnover, but also with a stimulation of the osteoblastic lineage leading to a more pronounced gain in bone density compared to other BPs.

## Figures and Tables

**Table 1:** Anthropometric, densitometric, and biochemical data of the study population.

Parameter	Baseline Values (Mean ± SD)	12 Months Values (Mean ± SD)	*p* Value
Age (years)	62 ± 11		
Weight (kg)	63 ± 10	-	-
height (m)	1.54 ± 0.06	-	-
BMI (kg/m^2^)	26 ± 5		
Lumbar BMD (g/cm^2^)	0.710 ± 0.128	0.76 ± 0.11	0.045
LumbarT-score (SD)	−2.9 ± 1.0	−2.5 ± 0.97	0.001
Lumbar Z-score (SD)	−1.4 ± 1.2	−0.98 ± 1.25	0.001
Femoral neck BMD (g/cm^2^)	0.608 ± 0.08	0.6 ± 0.07	0.78
Femoral neck T-score (SD)	−2.2 ± 0.74	−2.2 ± 0.64	0.71
Femoral neck Z-score (SD)	−0.8 ± 0.8	0.74 ± 0.71	0.204
Total hip BMD (g/cm^2^)	0.728 ± 0.086	0.73 ± 0.08	0.15
Total hip T-score (SD)	−1.8 ± 0.71	−1.7 ± 0.71	0.006
Total hip Z-score (SD)	−0.7 ± 0.8	−0.57 ± 0.83	0.002
Creatinine (mg/dL)	0.73 ± 0.15	0.73 ± 0.17	0.255
s-Calcium (mg/dL)	9.2 ± 0.2	9.4 ± 0.3	0.014
Calcium/creatinine (mg/mg)	0.140 ± 0.095	0.09 ± 0.06	0.023
ALP (U/L)	79.8 ± 21.7	72.06 ± 30.1	0.026
CTX (ng/mL)	0.525 ± 0.206	0.21 ± 0.1	0.001
PTH (pg/mL)	59.2 ± 32.8	80 ± 32	0.002
25-OH vitamin D (ng/mL)	31.5 ± 20.5	33.23 ± 12.37	0.07

BMI, body mass index; BMD, bone mineral density; ALP, alkaline phosphatase; CTX, C terminal telopeptide; PTH, parathormon; 25-OH, 25*-*hydroxy*-*vitamin *D*.

**Table 2:** Cell phenotype of MSC-like after depletion.

Cluster Differentiation	Controls	Pre-Therapy	6 Months PT	12 Months PT
CD3	0%	0%	0%	0%
CD14	0.48 ± 0.06%	0.70% (±0.04)	0.60% (±0.60)	0.80% (±0.07)
CD19	0%	0%	0%	0%
CD45	2.30 ± 0.47%	1.89% (±0.30)	1.70% (±0.80)	1.90% (±0.50)
CD34	Low level	Low level	Low level	Low level

PT, post-therapy.
